# Supplementary figures and images for: Meta-analytic evidence that allelopathy may increase the success and impact of invasive grasses
Source: PeerJ. 2023 Feb 21;11:e14858. doi: 10.7717/peerj.14858 (PMC9951799; doi:10.7717/peerj.14858)

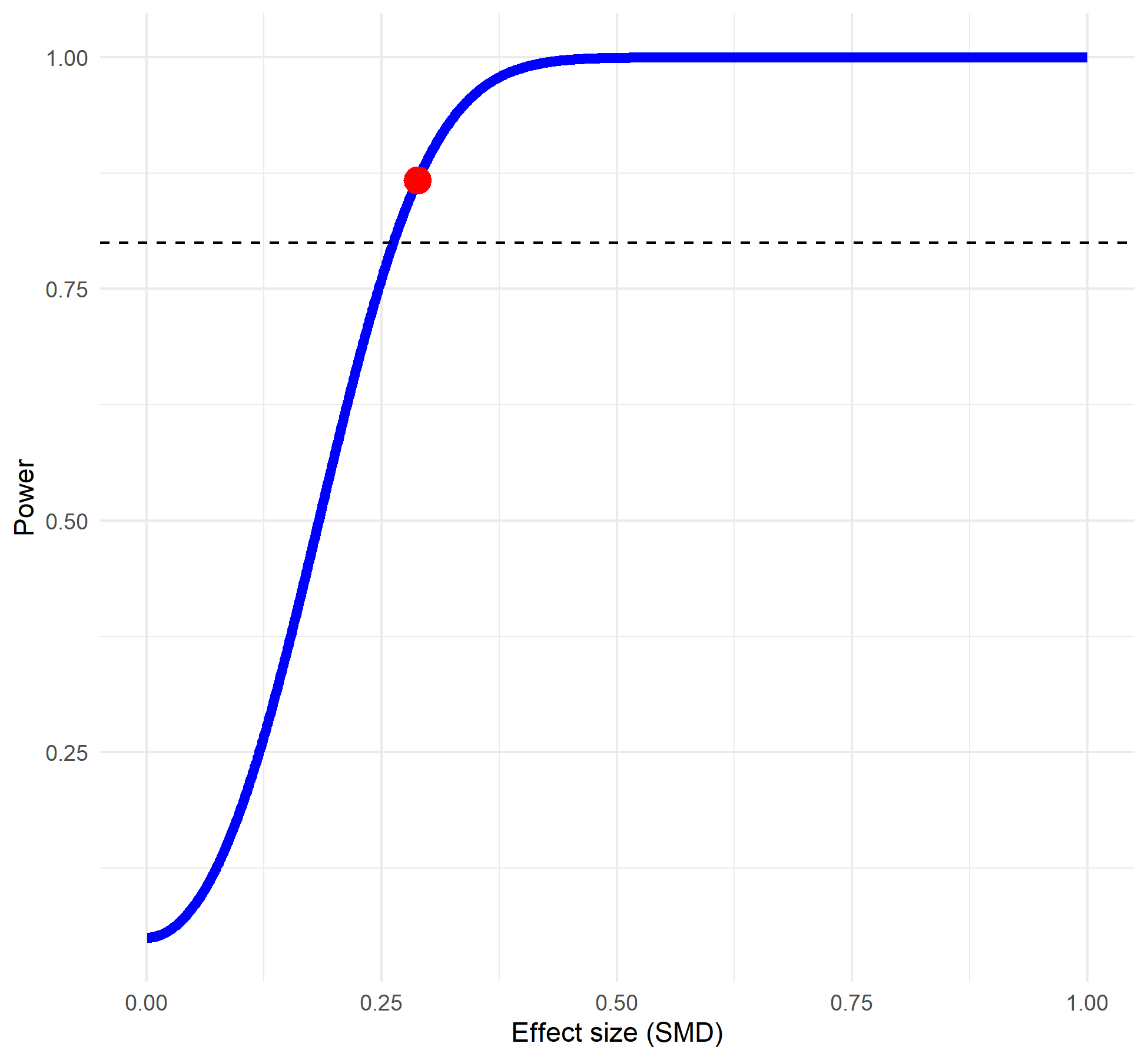

Supplement: Supplemental Information 1 — Heterogeneity was set to “high” and expected mean difference was set to 0.288 based on Zhang et al. (2020). Power was 86%. [file peerj-11-14858-s001.png]

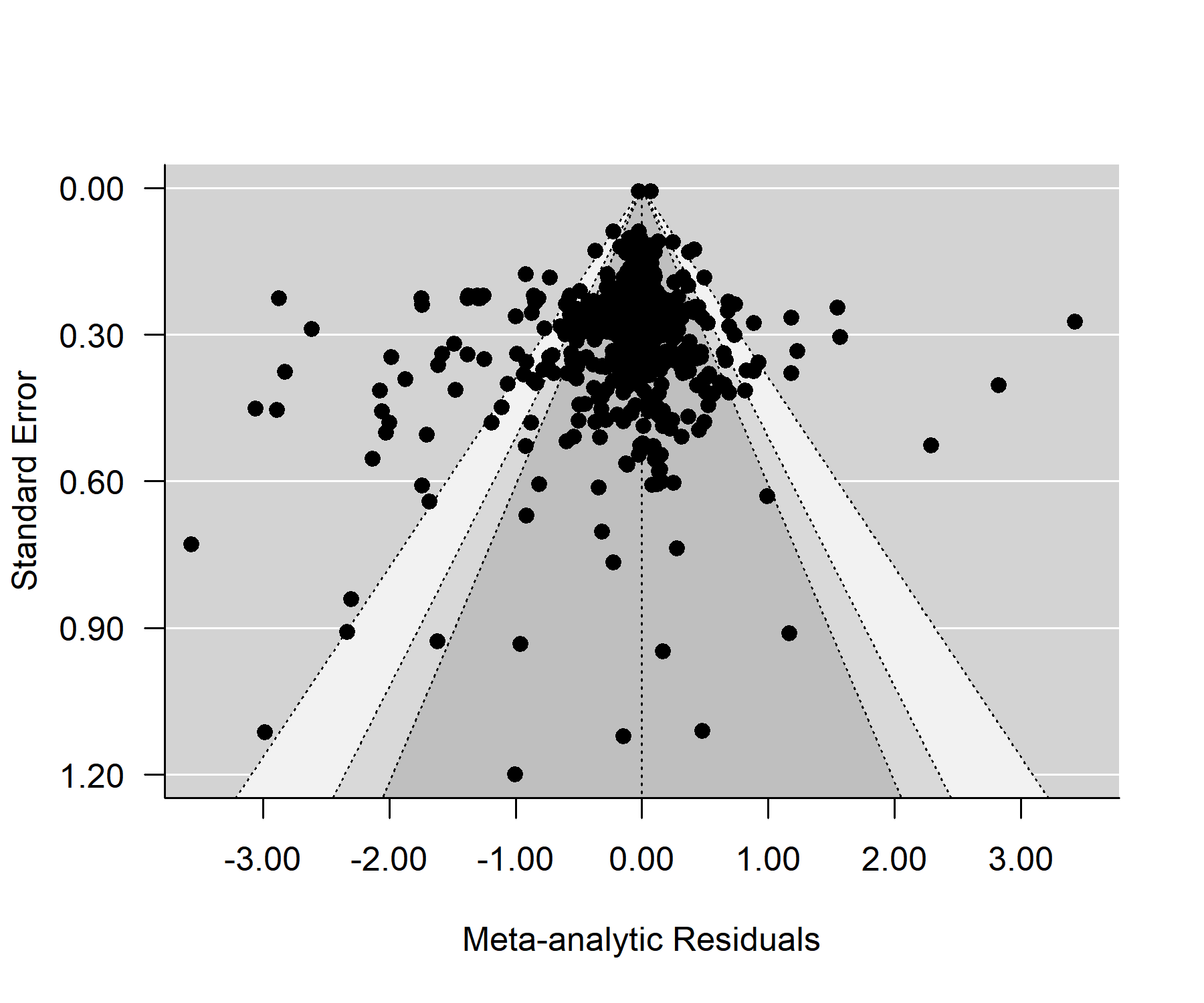

Supplement: Supplemental Information 2 — The spread of the points suggests publication bias, although Egger’s test does not provide strong statistical support for publication bias (the y-intercept 95% confidence interval includes zero). Points outlie the funnel on both the negative and positive side. Some extreme outliers were excluded for clarity of visual. [file peerj-11-14858-s002.png]

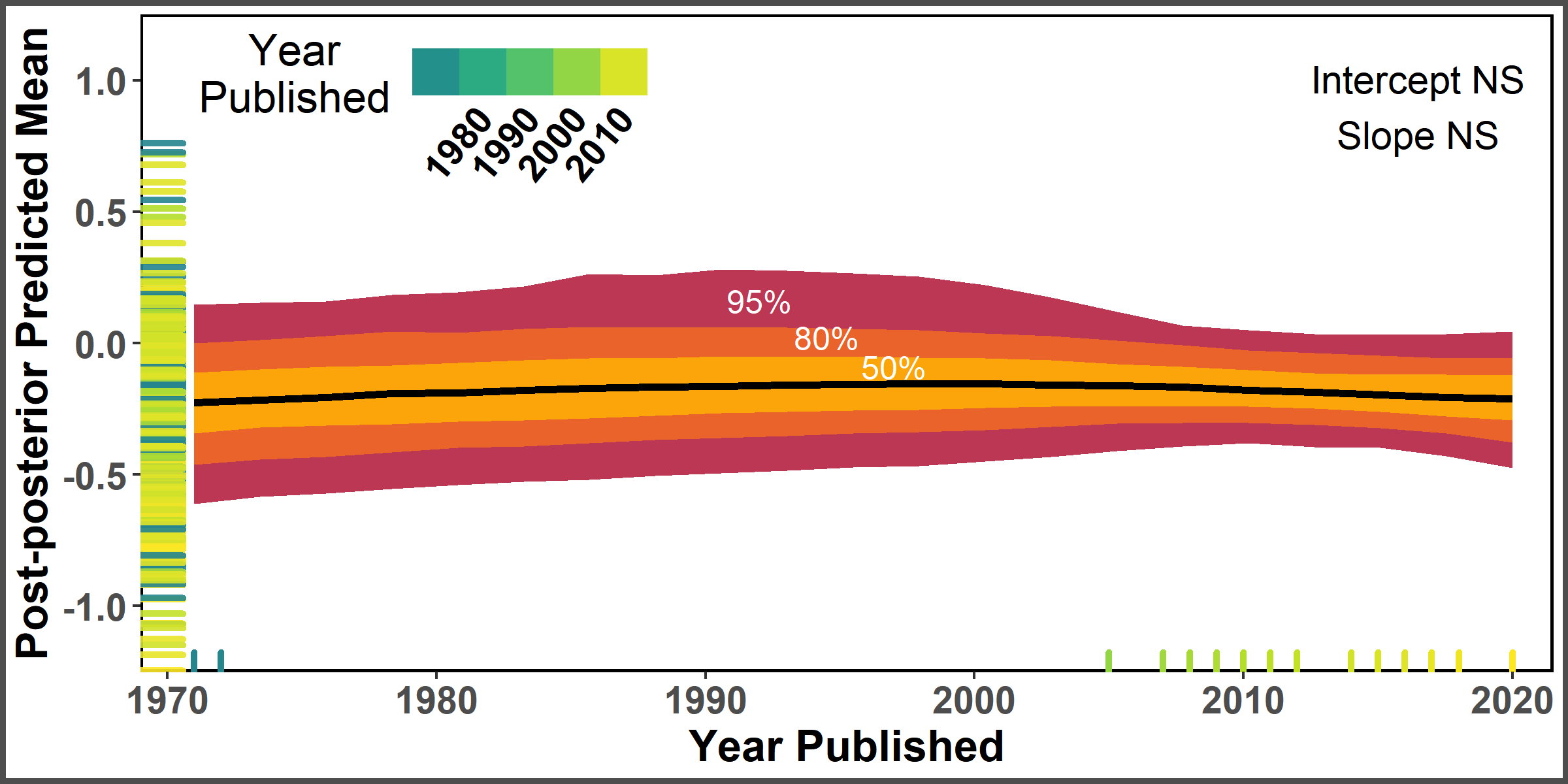

Supplement: Supplemental Information 3 — Rug plot on x and y axis represent distribution of observed effect sizes. [file peerj-11-14858-s003.png]

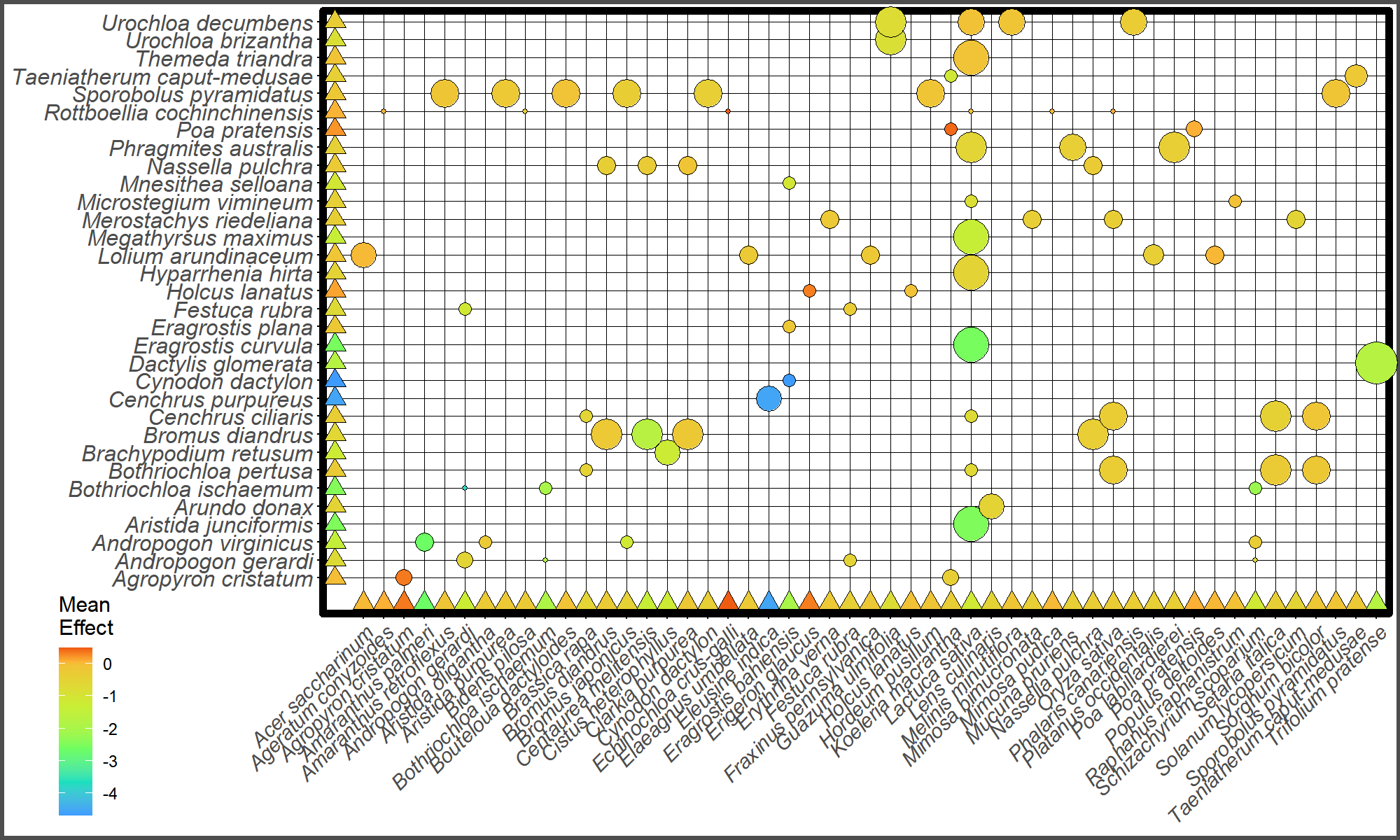

Supplement: Supplemental Information 4 — Balloon size represents frequency of each species pair. Triangle points represent the model predicted average effect size for each grass and recipient species. Color of balloons and triangles represents mean effect size. [file peerj-11-14858-s004.png]

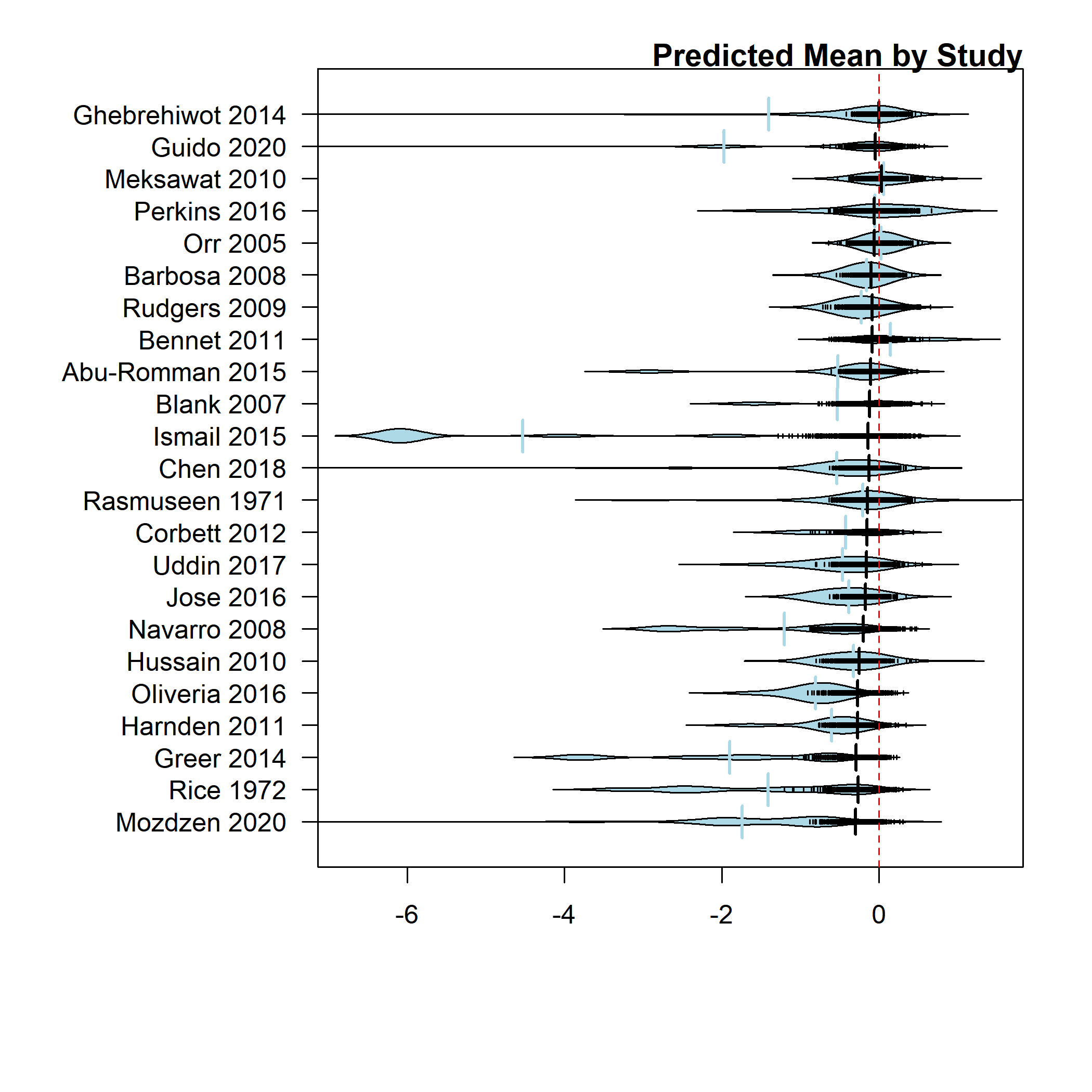

Supplement: Supplemental Information 5 [file peerj-11-14858-s005.png]

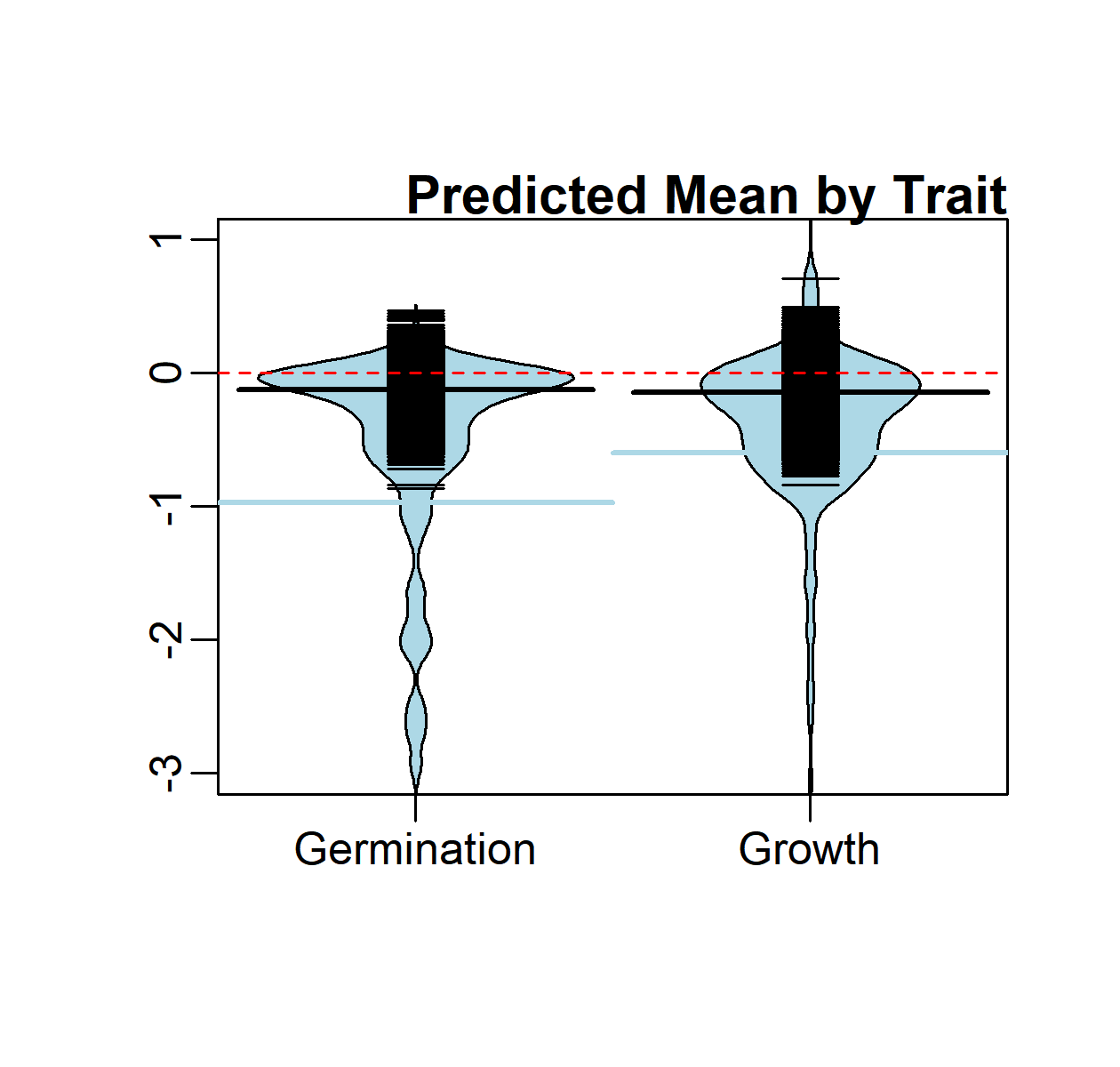

Supplement: Supplemental Information 6 [file peerj-11-14858-s006.png]

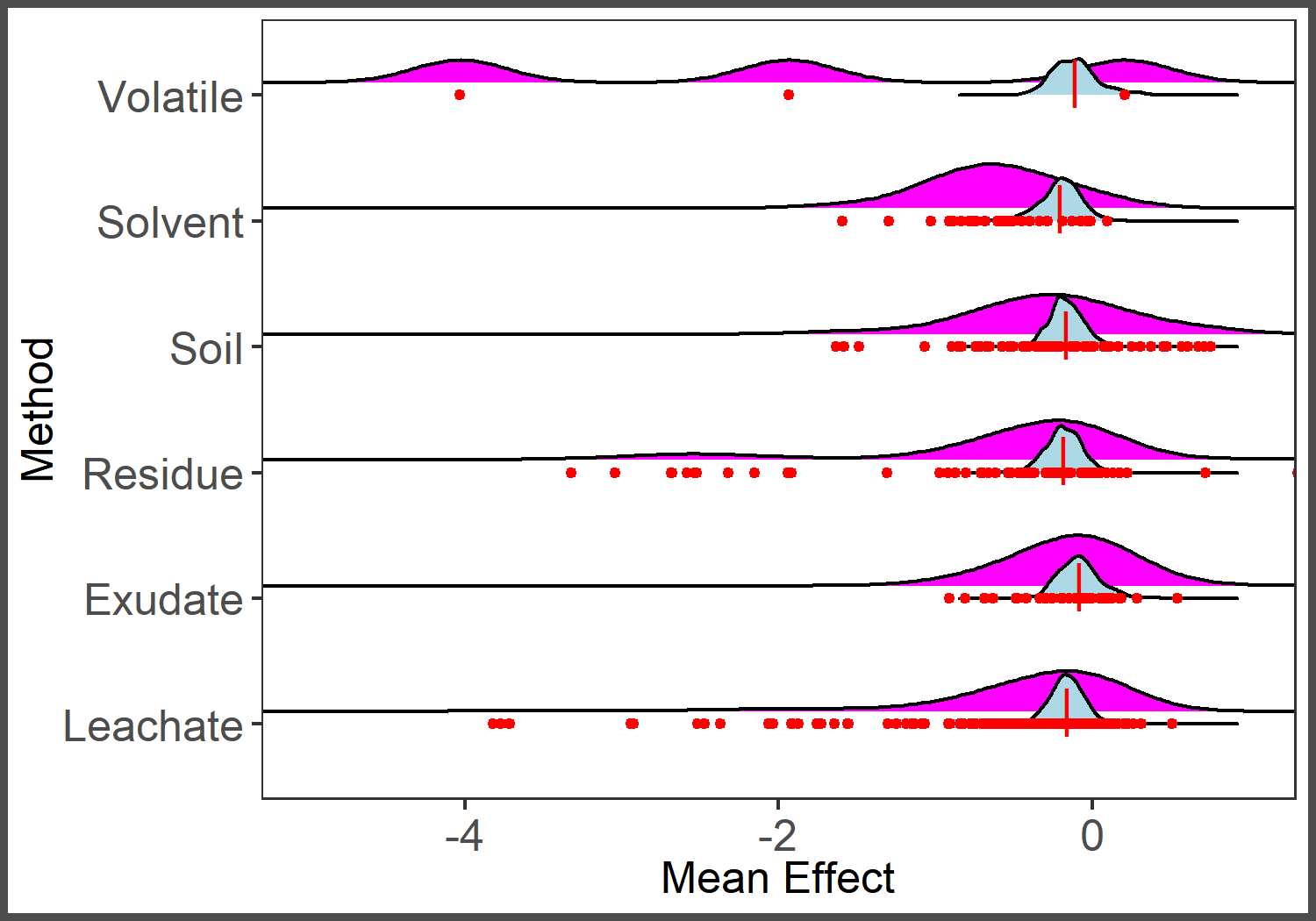

Supplement: Supplemental Information 7 — Points represent observed effect sizes (red) and long line represents mean of observed effect sizes (red). [file peerj-11-14858-s007.png]

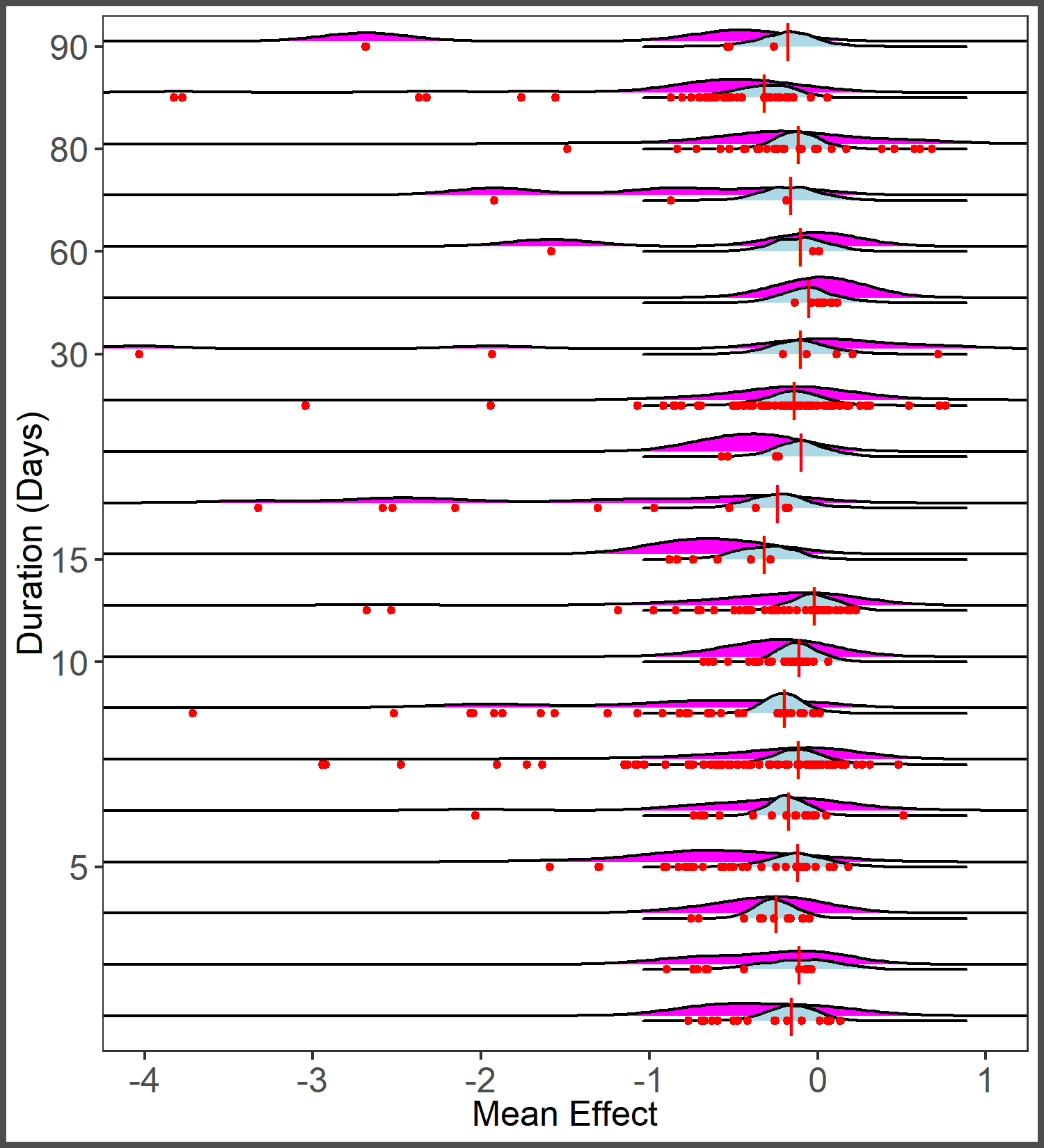

Supplement: Supplemental Information 8 — Points represent observed effect sizes (red) and long line represents mean of observed effect sizes (red). [file peerj-11-14858-s008.png]

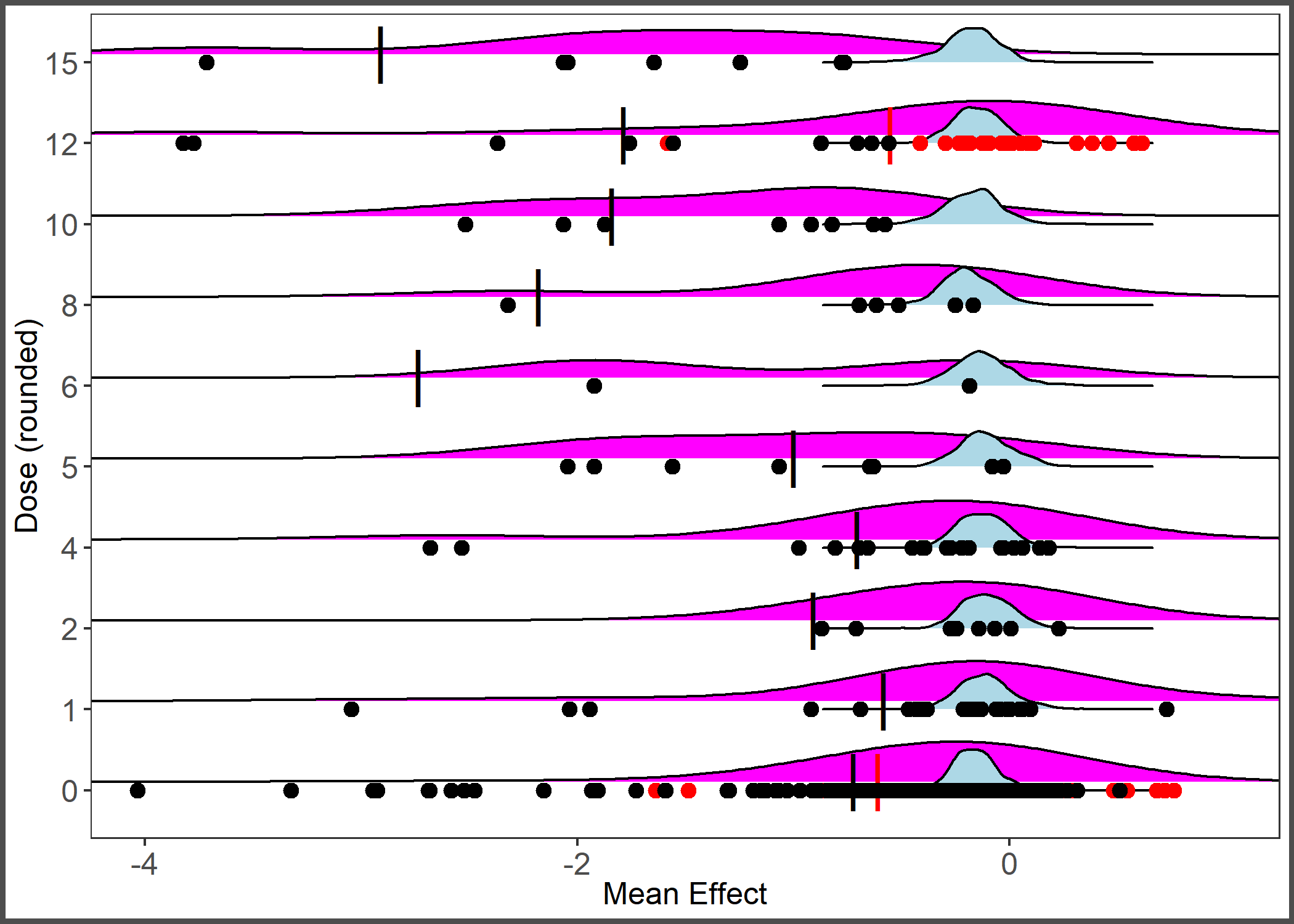

Supplement: Supplemental Information 9 — Points represent observed effect sizes by reported dose (black) and imputed dose (red). Long line represents mean with imputed dose values (red) and with only reported dose values (black). [file peerj-11-14858-s009.png]
