# Supplementary material for: Meta-analytic evidence that allelopathy may increase the success and impact of invasive grasses
Source: PeerJ. 2023 Feb 21;11:e14858. doi: 10.7717/peerj.14858 (PMC9951799; doi:10.7717/peerj.14858)
Supplement: Supplemental Information 10 [file peerj-11-14858-s010.docx]

| \| **Model** \| **LOO** \| **WAIC** \| \| --- \| --- \| --- \| \| m_full \| 0.000 \| 0.000 \| \| m_intercept \| -4.665 \| -4.616 \| |  |  |
| --- | --- | --- | --- | --- | --- | --- | --- | --- | --- | --- | --- |
|  |  |  |
|  |  |  |
