# Supplementary material for: Meta-analytic evidence that allelopathy may increase the success and impact of invasive grasses
Source: PeerJ. 2023 Feb 21;11:e14858. doi: 10.7717/peerj.14858 (PMC9951799; doi:10.7717/peerj.14858)
Supplement: Supplemental Information 11 [file peerj-11-14858-s011.docx]

Extended methods:

The first author performed the search strategy, and any disagreements were discussed between the two authors until a consensus was reached.

Studies were excluded when they did not fit into the scope of this meta-analysis, often because the study was testing allelopathy on grass, but not testing allelopathy by a grass. Additionally, studies were excluded if they provided insufficient data (lacking control data, lacking standard deviation or standard error, representing data only as percent of control). or did not fit into the accepted methodological approaches outlined in Zhang et al. (2020a). We also excluded one study conducted in a semi-controlled environment, since all other studies were conducted in controlled environments. The first author conducted the search and filtering process independently.

For each unique pair of species within a substudy, method and any other methodological variables we collected (color of plant tissue, source of plant tissue, dose or solvent), at most two pairs were extracted, one for germination, which was converted from percent germination to number of seeds germinated, or growth. For growth measurements, total growth was used when available. If total was not available, aboveground growth was used. If neither were available, belowground growth was used. If figures presented data over a range of time, the data for the last time point was used. For each study, data was extracted either from tables or figures, and the source of each pair was noted. Data were extracted from figures using Web Plot Digitizer (<https://automeris.io/WebPlotDigitizer/>).

The original dataset was used to generate a list of species (both recipient and allelopathy species). Names were checked against NBCI Taxonomy, and entered into a spreadsheet in the appropriate format for use with V.Phylomaker2. The species spreadsheet was turned into a phylogenetic tree, using the “Scenario 3” setting in the function phylo.maker. The phylogenetic tree was used to generate a distance matrix, using the function cophenetic. The distance matrix was used to fill the “pd” column with the appropriate value for each allelopathy species-recipient species pair. The dataset was then prepped for analysis.

First, an *a priori* power calculator was used (<https://raw.githubusercontent.com/MathiasHarrer/dmetar/master/R/power.analysis.R>). The analysis was set to predicted mean difference of 0.288, based on Zhang et al. (2020), study number of 23, and both n1 and n2 were set to 20. Confidence level was set to 90%, and heterogeneity was set to “high.” Finally, the prepped dataset was run through the final analysis.

All code used, as well as all initial and intermediate files are available at <https://doi.org/10.6084/m9.figshare.21720920>. Using the fully prepped dataset (supplementary_file_2.csv), phylogenetic tree (result3.nwk) and final analysis code (annotated_metaanalysis_code.RMD), one would be able to run through the final analysis, and produce all figures associated with this manuscript.
