# Supplementary material for: Meta-analytic evidence that allelopathy may increase the success and impact of invasive grasses
Source: PeerJ. 2023 Feb 21;11:e14858. doi: 10.7717/peerj.14858 (PMC9951799; doi:10.7717/peerj.14858)
Supplement: Supplemental Information 13 [file peerj-11-14858-s013.docx]

1. The rationale for conducting the systematic review / meta-analysis;
   1. A: The Poaceae family is unique in its invasive success, and in the presence of family specific (with exceptions which may be examples of convergent evolution) allelochemicals. With ecosystems worldwide being affected, work that establishes commonalities among invasive grasses can contribute to improvements in restoration that can have significant benefits for many communities. Restoration of grass-invaded areas is a timely-issue due to the association between invasive grasses and wildfires, with pose a threat not only to local ecosystems and communities, but also further contribute to the challenge of climate change. If we found that allelopathic capabilities were statistically significant in non-native grass-native recipient species pairs, then we can recommend that practioners consider specific allelopathy-informed restoration practices, with specifics on what information would be needed for implementing these practices successfully. If we found that allelopathic capabilities were not statistically significant in non-native grass-native recipient species pairs, this would suggest that other aspects of belowground change are major drivers of the soil legacies that makes restoration in grass-invaded areas so challenging. Either way, we would be able to make recommendations on practices practioners should consider, or future research needs, both of which would be meaningful contributions to the challenge of restoration in the UN Decade of Restoration.
2. The contribution that it makes to knowledge in light of previously published related reports, including other meta-analyses and systematic reviews
   1. A: Zhang et al. utilized a very broad scope, and in terms of growth forms, focused only on “woody” and “non-woody,” which they found no significant difference between. Our narrower scope means that our findings have clear and specific implications for practioners and for the critical issue of restoration. Furthermore, they took a very different approach to Bayesian modeling. They focused more heavily on fixed effects, while we included more random effects, which means that we had the model assume that different experimental variables, like dose, or study duration, would be associated with their own intercepts. We used smoothed terms, allowing the model to determine its own non-linear relationship, while Zhang et al. included hypothesized quadratic relationship, by including variables like dose and phylogenetic distance as squared terms, in addition to the variables alone. We used splines in our model, to split our variables into variables based on experimental design, versus variables based on inherent characteristics of the species. Finally, we used missing value imputation to make up for methodological approaches of assessing allelopathic abilities that make it challenging for scientists to determine what the dose of allelochemicals being tested might be. These methodological differences reflect advances in how proponents of Bayesian modeling think about constructing strong Bayesian models. The extent of the variance explained by our random variables shows the utility of Bayesian modeling for helping to account for the inherent variability in ecological phenomenon.
