# Supplementary material for: Meta-analytic evidence that allelopathy may increase the success and impact of invasive grasses
Source: PeerJ. 2023 Feb 21;11:e14858. doi: 10.7717/peerj.14858 (PMC9951799; doi:10.7717/peerj.14858)
Supplement: Supplemental Information 14 [file peerj-11-14858-s014.doc]

| **Checklist item** | **Item score** | **Sub-item number** | **Sub-item** | **Reported by authors?** | **Notes** |
| --- | --- | --- | --- | --- | --- |
| **Title and abstract** | **83%** | 1.1 | Identify the review as a systematic review, meta-analysis, or both | Yes | Title & Abstract Line 6 (Methods) |
|  |  | 1.2 | Summarise the aims and scope of the review | Yes | Abstract Methods |
|  |  | 1.3 | Describe the data set | Yes | Abstract Line 12-13 (final sentence of Methods) |
|  |  | 1.4 | State the results of the primary outcome | Yes | Abstract Results (first and second sentence) |
|  |  | 1.5 | State conclusions | Yes | Abstract Results (last two sentences) |
|  |  | 1.6 | State limitations | No | X |
| **Aims and questions** | **100%** | 2.1 | Provide a rationale for the review | Yes | Line 40-44 |
|  |  | 2.2 | Reference any previous reviews or meta-analyses on the topic | Yes | Line 65 |
|  |  | 2.3 | State the aims and scope of the review (including its generality) | Yes | Line 45-47 |
|  |  | 2.4 | State the primary questions the review addresses (e.g. which moderators were tested) | Yes | Line 47-55 |
|  |  | 2.5 | Describe whether effect sizes were derived from experimental and/or observational comparisons | Yes | Line 91-93 |
| **Review registration** | **0%** | 3.1 | Register review aims, hypotheses (if applicable), and methods in a time-stamped and publicly accessible archive and provide a link to the registration in the methods section of the manuscript. Ideally registration occurs before the search, but it can be done at any stage before data analysis. | No | X |
|  |  | 3.2 | Describe deviations from the registered aims and methods | No | X |
|  |  | 3.3 | Justify deviations from the registered aims and methods | No | X |
| **Eligibility criteria** | **100%** | 4.1 | Report the specific criteria used for including or excluding studies when screening titles and/or abstracts, and full texts, according to the aims of the systematic review (e.g. study design, taxa, data availability) | Yes | Line 67-71 and Figure 1 |
|  |  | 4.2 | Justify criteria, if necessary (i.e. not obvious from aims and scope) | Yes | Line 68-69 |
| **Finding studies** | **100%** | 5.1 | Define the type of search (e.g. comprehensive search, representative sample) | Yes | Line 58 |
|  |  | 5.2 | State what sources of information were sought (e.g. published and unpublished studies, personal communications) | Yes | Line 59 |
|  |  | 5.3 | Include, for each database searched, the exact search strings used, with keyword combinations and Boolean operators | Yes | Line 59-60 |
|  |  | 5.4 | Provide enough information to repeat the equivalent search (if possible), including the timespan covered (start and end dates) | Yes | Line 61-63 |
| **Study selection** | **100%** | 6.1 | Describe how studies were selected for inclusion at each stage of the screening process (e.g. use of decision trees, screening software) | Yes | Figure 1 |
|  |  | 6.2 | Report the number of people involved and how they contributed (e.g. independent parallel screening) | Yes | Supplemental File 1- Extended Methods, paragraph 1  Also in PeerJ prompts |
| **Data collection process** | **100%** | 7.1 | Describe where in the reports data were collected from (e.g. text or figures) | Yes | Supplemental File 2 (in the dataset itself) |
|  |  | 7.2 | Describe how data were collected (e.g. software used to digitize figures, external data sources) | Yes | Supplemental File 1- Extended Methods Paragraphs 2 and 3 |
|  |  | 7.3 | Describe moderator variables that were constructed from collected data (e.g. number of generations calculated from years and average generation time) | Yes | Line 79-89 and Supplemental File 1 Extended Methods |
|  |  | 7.4 | Report how missing or ambiguous information was dealt with during data collection (e.g. authors of original studies were contacted for missing descriptive statistics, and/or effect sizes were calculated from test statistics) | Yes | Line 96-104 |
|  |  | 7.5 | Report who collected data | Yes | Supplemental File 1- Extended Methods paragraph 1  Also in PeerJ prompts |
|  |  | 7.6 | State the number of extractions that were checked for accuracy by co-authors | not applicable | X |
| **Data items** | **100%** | 8.1 | Describe the key data sought from each study | Yes | Line 91-93 |
|  |  | 8.2 | Describe items that do not appear in the main results, or which could not be extracted due to insufficient information | Yes | Line 79-87 and Supplemental File 1 |
|  |  | 8.3 | Describe main assumptions or simplifications that were made (e.g. categorising both 'length' and 'mass' as 'morphology') | Yes | Line 79-96 |
|  |  | 8.4 | Describe the type of replication unit (e.g. individuals, broods, study sites) | Yes | Supplemental File 1- Extended Methods |
| **Assessment of individual study quality** | **100%** | 9.1 | Describe whether the quality of studies included in the systematic review or meta-analysis was assessed (e.g. blinded data collection, reporting quality, experimental versus observational) | Yes | Line 91-93 |
|  |  | 9.2 | Describe how information about study quality was incorporated into analyses (e.g. meta-regression and/or sensitivity analysis) | Yes | Line 140-145 |
| **Effect size measures** | **100%** | 10.1 | Describe effect size(s) used | Yes | Line 91-93 |
|  |  | 10.2 | Provide a reference to the equation of each calculated effect size (e.g. standardised mean difference, log response ratio) and (if applicable) its sampling variance | Yes | Line 93 |
|  |  | 10.3 | If no reference exists, derive the equations for each effect size and state the assumed sampling distribution(s) | not applicable | X |
| **Missing data** | **100%** | 11.1 | Describe any steps taken to deal with missing data during analysis (e.g. imputation, complete case, subset analysis) | Yes | Line 96-104 |
|  |  | 11.2 | Justify the decisions made to deal with missing data | Yes | Line 102-104 |
| **Meta-analytic model description** | **100%** | 12.1 | Describe the models used for synthesis of effect sizes | Yes | Line 119-135 |
|  |  | 12.2 | The most common approach in ecology and evolution will be a random-effects model, often with a hierarchical/multilevel structure. If other types of models are chosen (e.g. common/fixed effects model, unweighted model), provide justification for this choice | Yes | Line 119-135 |
| **Software** | **100%** | 13.1 | Describe the statistical platform used for inference (e.g. R) | Yes | Line 108 |
|  |  | 13.2 | Describe the packages used to run models | Yes | Line 108-117 |
|  |  | 13.3 | Describe the functions used to run models | Yes | Line 108-109 |
|  |  | 13.4 | Describe any arguments that differed from the default settings | Yes | Line 109-110 |
|  |  | 13.5 | Describe the version numbers of all software used | Yes | Line 108 |
| **Non-independence** | **100%** | 14.1 | Describe the types of non-independence encountered (e.g. phylogenetic, spatial, multiple measurements over time) | Yes | Line 111-113;127-132 |
|  |  | 14.2 | Describe how non-independence has been handled | Yes | Line 111-113;127-132 |
|  |  | 14.3 | Justify decisions made | Yes | Line 124-126 |
| **Meta-regression and model selection** | **100%** | 15.1 | Provide a rationale for the inclusion of moderators (covariates) that were evaluated in meta-regression models | Yes | Line 124-126 |
|  |  | 15.2 | Justify the number of parameters estimated in models, in relation to the number of effect sizes and studies (e.g. interaction terms were not included due to insufficient sample sizes) | No | Only 3 fixed effects were used |
|  |  | 15.3 | Describe any process of model selection | Yes | Line 136-139 |
| **Publication bias and sensitivity analysis** | **100%** | 16.1 | Describe assessments of the risk of bias due to missing results (e.g. publication, time-lag, and taxonomic biases) | Yes | Line 142-145 |
|  |  | 16.2 | Describe any steps taken to investigate the effects of such biases (if present) | Yes | Line 142-145 |
|  |  | 16.3 | Describe any other analyses of robustness of the results, e.g. due to effect size choice, weighting or analytical model assumptions, inclusion or exclusion of subsets of the data, or the inclusion of alternative moderator variables in meta-regressions | Yes | Line 142-145 |
| **Clarification of post hoc analyses** | **100%** | 17.1 | When hypotheses were formulated after data analysis, this should be acknowledged. | Yes | Hypothesis were formed prior to analysis |
| **Metadata, data, and code** |  | 18.1 | Share metadata (i.e. data descriptions) | Yes | Metadata included in dataset |
|  |  | 18.2 | Share data required to reproduce the results presented in the manuscript | Yes | Dataset uploaded |
|  |  | 18.3 | Share additional data, including information that was not presented in the manuscript (e.g. raw data used to calculate effect sizes, descriptions of where data were located in papers) | Yes | Included in dataset |
|  |  | 18.4 | Share analysis scripts (or, if a software package with graphical user interface (GUI) was used, then describe full model specification and fully specify choices) | Yes | Line 108-135 |
| **Results of study selection process** | **100%** | 19.1 | Report the number of studies screened | Yes | Line 68 |
|  |  | 19.2 | Report the number of studies excluded at each stage of screening | Yes | Figure 1 |
|  |  | 19.3 | Report brief reasons for exclusion from the full text stage | Yes | Figure 1, Line 67-72 |
|  |  | 19.4 | Present a Preferred Reporting Items for Systematic Reviews and Meta-Analyses (PRISMA)-like flowchart (www.prisma-statement.org). | Yes | Figure 1 |
| **Sample sizes and study characteristics** | **60%** | 20.1 | Report the number of studies and effect sizes for data included in meta-analyses | Yes | Line 71 |
|  |  | 20.2 | Report the number of studies and effect sizes for subsets of data included in meta-regressions | Yes | Line 71 |
|  |  | 20.3 | Provide a summary of key characteristics for reported outcomes (either in text or figures; e.g. one quarter of effect sizes reported for vertebrates and the rest invertebrates) | No | X |
|  |  | 20.4 | Provide a summary of limitations of included moderators (e.g. collinearity and overlap between moderators) | Yes | Line 108-135 |
|  |  | 20.5 | Provide a summary of characteristics related to individual study quality (risk of bias) | No | X |
| **Meta-analysis** | **100%** | 21.1 | Provide a quantitative synthesis of results across studies, including estimates for the mean effect size, with confidence/credible intervals | Yes | Line 149-152 |
| **Heterogeneity** |  | 22.1 | Report indicators of heterogeneity in the estimated effect (e.g. I2, tau2 and other variance components) | Yes | Line 152-157 |
| **Meta-regression** |  | 23.1 | Provide estimates of meta-regression slopes (i.e. regression coefficients) and confidence/credible intervals | Yes | Line 164-184 |
|  |  | 23.2 | Include estimates and confidence/credible intervals for all moderator variables that were assessed (i.e. complete reporting) | Yes | Results section |
|  |  | 23.3 | Report interactions, if they were included | not applicable | X |
|  |  | 23.4 | Describe outcomes from model selection, if done (e.g. R2 and AIC) | Yes | Table S1 |
| **Outcomes of publication bias and sensitivity analysis** | **100%** | 24.1 | Provide results for the assessments of the risks of bias (e.g. Egger's regression, funnel plots) | Yes | Line 157-161 Figure S2 |
|  |  | 24.2 | Provide results for the robustness of the review's results (e.g. subgroup analyses, meta-regression of study quality, results from alternative methods of analysis, and temporal trends) | Yes | Line 161-162 |
| **Discussion** | **83%** | 25.1 | Summarise the main findings in terms of the magnitude of effect | Yes | Line 191-192;  Line 213-215;  Line 229-231 |
|  |  | 25.2 | Summarise the main findings in terms of the precision of effects (e.g. size of confidence intervals, statistical significance) | Yes | Line 191-192;  Line 213-215;  Line 229-231 |
|  |  | 25.3 | Summarise the main findings in terms of their heterogeneity | Yes | Line 232-242 |
|  |  | 25.4 | Summarise the main findings in terms of their biological/practical relevance | Yes | Line 243-285 |
|  |  | 25.5 | Compare results with previous reviews on the topic, if available | No | Zhang et al. results used to inform power analysis and priors |
|  |  | 25.6 | Consider limitations and their influence on the generality of conclusions, such as gaps in the available evidence (e.g. taxonomic and geographical research biases) | Yes | Line 201-226 |
| **Contributions and funding** | **100%** | 26.1 | Provide names, affiliations, and funding sources of all co-authors | Yes | Title page |
|  |  | 26.2 | List the contributions of each co-author | Yes | Within PeerJ authorship prompts |
|  |  | 26.3 | Provide contact details for the corresponding author | Yes | Within PeerJ prompts |
|  |  | 26.4 | Disclose any conflicts of interest | Yes | Within PeerJ prompts |
| **References** | **100%** | 27.1 | Provide a reference list of all studies included in the systematic review or meta-analysis | Yes | In cited references |
|  |  | 27.2 | List included studies as referenced sources (e.g. rather than listing them in a table or supplement) | Yes | Line 72-78 |
